# Caring relationships in times of crisis: the role of families during the Covid-19 pandemic

**DOI:** 10.3389/fsoc.2025.1725120

**Published:** 2026-02-06

**Authors:** Isabella Crespi, Marta Scocco

**Affiliations:** Department of Education, Cultural Heritage and Tourism, University of Macerata, Macerata, Italy

**Keywords:** Covid-19, family, generations, informal caregiving, relationships

## Abstract

**Introduction:**

Caregiving is shaped by social system factors that influence the availability of formal and informal support and represents a key life-course experience linked to ageing and family roles. Intra-family relationships and the exchange of emotional and practical support are crucial for older adults’ well-being, yet remain underexplored in relation to the Covid-19 pandemic. At the European level, individuals aged 65+ with long-term care needs and their informal caregivers were among the most affected population groups.

**Methods:**

This study analyses open-access data from the Survey of Health, Ageing and Retirement in Europe (SHARE) to investigate the Italian context. The work examines pandemic-related changes in family care strategies, relational dynamics, and the adoption of digital tools to support informal caregiving.

**Results:**

Findings confirm the substantial increase in caregiver burden and care recipients’ vulnerability associated with the unintended consequences of Covid-19 epidemiological control measures. Data show disruptions in care arrangements and heightened emotional and practical strain within families.

**Discussion:**

Alongside challenges, results highlight underexplored functional aspects of family care practices, particularly the supportive role of new technologies. Digital tools emerged as a relevant resource for sustaining caregiving and mitigating relational and organisational breakdowns, suggesting the need to further integrate technological support into informal care strategies.

## 1 Introduction

A growing need for care and assistance is emerging in a European demographic context characterised by progressive population ageing, a trend expected to persist in the coming decades. By 2025, the percentage of people aged 65 or older in the European Union is estimated to reach approximately 21% of the total population (Eurostat, 2025; Daly and Lewis, 2000; Daly and Rake, 2003). In Southern European countries in particular, welfare systems continue to rely heavily on family support and informal caregiving practices.

Against this background, this study examines how family-based care practices and intergenerational relationships in Italy were reshaped during the Covid-19 pandemic, with particular attention to informal caregiving arrangements and the role played by digital technologies. Using SHARE data, the article aims to contribute to the understanding of how familistic welfare systems responded to the crisis and how existing inequalities—especially gendered and digital—were reinforced or transformed.

In this conceptual framework, families increasingly express a need for social protection to meet care demands that have surpassed their internal resources. Traditionally intrinsic to familial roles and responsibilities, these demands have progressively shifted towards forms of external outsourcing. Consequently, caregiving obligations are being realigned among the family unit, the state, and the market, as outlined by Daly and Lewis (2000). These dynamics are strongly influenced by complex societal variables, including political factors that directly affect the conditions of caregivers and care recipients.

From a theoretical perspective, caregiving cannot be understood solely as a functional response to demographic ageing or welfare retrenchment. Drawing on Tronto’s (1993, 2013) ethics of care, care can be conceptualised as a moral and political practice embedded in asymmetries of power, responsibility, and social recognition. Tronto’s framework highlights how care work has historically been undervalued due to its association with women and the private domestic sphere, a dynamic that remains particularly salient in familistic welfare regimes such as Italy’s.

Complementing this perspective, Janet Finch’s analysis of the negotiated nature of family responsibilities (Finch and Mason, 1993) provides an analytical lens for understanding caregiving roles as shaped by implicit norms and expectations within kinship networks rather than by formal obligations alone. From this viewpoint, the feminisation of care emerges not merely as an empirical pattern, but as the outcome of deeply rooted cultural norms and institutional arrangements that assign caring responsibilities disproportionately to women.

Furthermore, how societies care for their most vulnerable members reflect fundamental cultural values, with the family playing a central role in socialisation processes, role definition, and the construction of intergenerational bonds. With the outbreak of the Covid-19 pandemic in early 2020, epidemiological control measures—such as mobility restrictions and physical distancing—significantly affected the wellbeing of European citizens, profoundly limiting the ability to provide care beyond the immediate household. In the Italian context, the health emergency highlighted the crucial role of family ties and interpersonal relations in providing care and support.

While economic, political, and health dimensions of the pandemic have been widely examined in comparative research, informal care practices based on intergenerational relationships have received comparatively less attention (Bramanti, 2023; Lynch et al., 2021). Addressing this gap, the present study focuses on the adaptations, strategies, and tools families use to cope with the crisis, with the aim of informing future welfare policies and care systems.

The article is structured as follows. After this introduction, the next sections outline the theoretical framework and review relevant literature on family-based care and the impact of the pandemic. The subsequent section presents the data, methodology, and analytical strategy. The final empirical section focuses on the Italian context, discussing changes in family dynamics and care practices, which are then synthesised in the conclusions.

## 2 Ageing and increasing care needs among Italian families

The phenomenon of population ageing appears to be unavoidable in almost all developed countries (Eurostat, 2023, 2025). This process is characterised by its intensity, which is the result of a combination of low birth rates, high life expectancy, migration flows, social and demographic policies, and changes in the labour market. Europe is facing an increasing demand for policies to support its elderly population, with economic challenges related to pension support, healthcare sustainability, and maintaining a productive workforce. However, it is also the effect of an age structure inherited from the past: that is, the presence of cohorts formed in high birth-rate eras, sometimes accurate ‘baby booms’, which are gradually reaching the top of the age pyramid. These dynamics are of considerable relevance in the Italian context, where, as of 1 January 2023, the population over 65 amounts to 14.177 million individuals, representing 24.1 per cent of the total population (Istat - Istituto Nazionale di Statistica, 2023). Demographic projections in Italy paint a potential crisis scenario. The resident population shows a downward trend, decreasing from 59.6 million on 1 January 2020 to 58 million in 2030, 54.1 million in 2050, and finally 47.6 million in 2070. These figures indicate an expected ratio of 1–3 between young and old by 2050. As in most European countries, Italy’s demographic pyramid from 1950 to 2030 tends to reverse, with an increasing number of people over 65 accompanied by a substantial decrease in the younger members of the population. According to estimates, in 2041 the population over 80 will increase by 35.2 percent compared to 2021, exceeding 6 million; that of the over-90s will reach 1.4 million (+ 69.4% over 2021) (Istat - Istituto Nazionale di Statistica, 2023).

These are indicative statistics that, however, pose several questions about the future capacity of institutions to cope with an unfamiliar demographic situation, in that no major country has ever experienced it in such proportions, where the need for care and assistance is bound to grow (Ascoli, 2020). Despite the recent spread of an active and prosperous ageing paradigm (Walker and Zaidi, 2016), this trend is associated with a progressive and inevitable decline in people’s state of health: with advancing age grows the onset of chronic and degenerative diseases, which limits the individual’s autonomy and intensifies his or her need for care (Bergmann et al., 2019; Bergmann and Wagner, 2021; Naldini, 2021). These can be provided formally, i.e., by qualified and paid staff, or informally, by those who dedicate their time to assist the dependent person.

Within the Italian context, this phenomenon assumes a distinctive character attributed to the intricate configuration of the welfare system. Italy is renowned for its exceptional legislative ‘inertia’, as cogently articulated by Pavolini and Ranci (2008). Older people’s care policies within the country show marked fragmentation and are situated within an institutional framework that places a substantial premium on relatives, friends or neighbours’ familial responsibilities, as meticulously elucidated by Millar and Warman (1996). From a more comprehensive perspective, the Italian Long-Term Care (LTC) system tends to prioritise financial incentives for individuals with frailties over establishing formal care services. Consequently, the system faces a significant deficit in formal services designed for the frail and dependent population. In fact, Italy claims the highest share in Europe of households reporting systemic inadequacies in professional care services, as substantiated by European Commission (2021b).

Additionally, the workforce within the LTC sector remains notably constricted, with a workforce numbering only 260,000 individuals, according to European Commission (2021a). The Long-Term Care system in Italy can only respond to one out of three people in need, which significantly restricts access to formal care for a considerable portion of the population. So, two-thirds of the older population who do not receive a response from the public welfare system seek alternatives to meet their care needs (Fosti and Notarnicola, 2018). The utilisation of informal care becomes the primary recourse for economically disadvantaged families. Owing to the family unit’s paramount role, the LTC system’s institutional configuration in Italy heavily relies on informal caregiving. More than 50 per cent of older people receive help from family members, 17 per cent from paid staff, and 6.4 per cent from other individuals (friends, voluntary associations, etc.). Among the older people with severe personal care difficulties (approximately 1.5 million persons), 84.4 percent report receiving help from family members (cohabiters or not). This percentage comprises 51.9 per cent who receive help exclusively from family members and 32.5 per cent who are supported by other persons, such as formal caregivers, paid staff, and home assistants appointed by public or private institutions (Istat - Istituto Nazionale di Statistica, 2021).

The term used to designate informal care providers is ‘caregiver’ (Brenna, 2018; Parker, 2020). In sociology, the concept of “caregiver” is linked not only to the practical aspects of caregiving but also to the social, cultural, and economic dynamics that influence who takes on the caregiver role and how this affects the lives of those involved. Caregivers can be family members, but they can also be professionals (such as home assistants or nurses) or individuals who provide care voluntarily.

The concept of ‘care’ in this context is considered in a multidimensional perspective (Fraser, 1996; Fine, 2005). The international literature distinguishes two different categories of activities in which the older person may experience limitations: Activities of Daily Living (ADL) and Instrumental Activities of Daily Living (IADL). The former refers to routine activities, such as getting out of bed, washing, dressing, and eating, that require constant assistance, and to a strong personal bond with the older person. The second are less demanding in terms of continuity and emotional stress but are still necessary for the older person to live independently at home; these include housework, dealing with banks and institutions, help with shopping, and adherence to medical prescriptions (Brenna, 2018). In this work, the concept of caring is analysed as a complex phenomenon that involves emotional, relational, ethical, social, and material aspects. It is not simply reduced to an individual gesture or a practical activity (such as providing assistance), but is interpreted as a complex social practice that reflects and reproduces power dynamics, inequalities, and cultural structures.

As in all Southern European countries and Italy, care management of non-self-sufficient persons is primarily delegated to the family. The welfare system is strongly supported by families, which provide care and assistance to many older individuals (Istat - Istituto Nazionale di Statistica, 2020; Melchiorre et al., 2022).

Within informal caregiving, Italy occupies a mid-range position in terms of the percentage of individuals involved in this role, according to data from European Commission (2018). Self-reported caregivers constitute 17 percent of the total population, with 19 percent women and 16 percent men. A noteworthy gender disparity becomes evident when focusing on the 45–64 age group, where the proportion of female caregivers (approximately 26%) significantly surpasses that of male caregivers (around 17%) (European Commission, 2021a). This scenario reveals a pronounced gender-based disparity (Naldini et al., 2016), as women disproportionately shoulder the burden of care work, as substantiated by UNECE – Working Group on Ageing (2019) and research by Le Bihan et al. (2019). Specifically, regarding older people’s care, the category most affected is that of female daughters (Brenna, 2018), who are often squeezed between responsibilities towards their not-yet-independent children and caring for their older parents and therefore defined, together with their peers, as the ‘sandwich generation’ (Hämäläinen and Tanskanen, 2021; Macchioni, 2020).

When considering the entire population within specific age brackets, informal caregivers constitute one-fifth of those aged 35–64 and 18 percent of individuals over the age of 65. In contrast, the percentage of caregivers within the 18–34 age group is notably lower, standing at 12 percent. In the perspective of the active population, specifically those aged 18–64, informal caregivers account for 10.5 percent (European Commission, 2018). Concerning employment status, the proportion of caregivers who are employed (5.8%) is slightly higher than those who are not employed (4.7%). These statistics position Italy in close alignment with the European average. In fact, the European percentage of informal caregivers within the overall working population is marginally higher, at 12.2 percent, compared to Italy.

Conversely, the percentage of caregivers who are not employed in Italy is very similar to the European average, at 4.8 per cent, whereas the European figure for employed caregivers is higher, at 7.5 per cent. The Italian situation reflects a broader trend, where most countries exhibit a lower incidence of informal caregiving within the working-age population (10.5%) compared to the total population (17%). This phenomenon is primarily attributed to the substantial presence of caregivers aged 65 and above, many of whom have retired from the workforce.

As discussed, in the Italian context, relatives are mainly responsible for assisting older people who need help with daily personal or household care activities (Bramanti, 2023). Family is increasingly at the centre of a dense network of relations between genders and generations, between peers, friends and neighbours, work colleagues, and members of associations and groups. The ability to care for family members is one of the fundamental aspects characterising family relationship dynamics. The strengthening of family ties is also achieved through caring, a specific mode that mobilises loyalty and generosity expectations among family members (Bramanti and Pavesi, 2015; Albertini and Tosi, 2022). From a cultural point of view, the Italian family, through the process of socialisation, continues to exert considerable influence in shaping caring roles, teaching skills of care to its members and fostering attitudes of responsibility and obligation to care towards both relatives and individuals outside the extended family unit (Brenna, 2021; Patterson and Margolis, 2019). Caring implies, through taking responsibility for the other’s needs, the creation and consolidation of a social relationship based on reciprocity and generosity (Bramanti and Pavesi, 2015). Inter- and intragenerational relationships are recognised as crucial in maintaining the wellbeing of the most vulnerable individuals (Barrett et al., 2025). A key aspect of these relationships is the exchange of both emotional and instrumental social support. However, relatively little is known about how this exchange of support changes in the context of widespread disruption (Derrer-Merk et al., 2024).

## 3 The Covid-19 pandemic in Italy: effects on family care relationships

The pandemic outbreak led to a rapid and profound shift in global society, accompanied by undesirable consequences. This event, one of the most severe economic shocks, generated changes with lasting impacts on employment and working conditions, growth and development experiences, education and learning, the provision and receipt of care (not limited to the health sphere), and social practices. It generally influenced people’s habits and life plans (Bergmann and Wagner, 2021; Naldini, 2021).

Like many other countries, Italy faced a health emergency in early 2020. On 21 February 2020, the Italian government introduced restrictive lockdown measures in the two northern regions of Lombardy and Veneto. These measures were subsequently extended to the entire national territory on 9 March 2020. Though these actions proved effective in flattening the epidemiological curve, Italian families have been overwhelmed by numerous and profound changes that also highlighted their resilience, their ability to cope with tasks and challenges, not least that of caring practices (Di Nicola and Ruspini, 2020; Lagomarsino et al., 2020; Mazzucchelli et al., 2020; Crespi et al., 2023), in a context in which social contacts, interactions, the possibility of providing care to others and receiving care from people outside one’s home dimension have been considerably reduced (Vergauwen et al., 2022).

Undeniably, catastrophic events often bring preexisting vulnerabilities into sharper focus. During the pandemic, it was possible to identify a specific population segment that was particularly affected by the emergency, both medically, psychologically, and relationally: older adults and the frail elderly (Nanetti et al., 2020). This observation supports the multidimensional nature of the care concept, as discussed in the previous paragraph, in which it is restrictive to include only the more practical issues of care, and instead, it is essential to recognise the emotional value that is reinforced through care relations, but is also a foundational part of it, especially in informal caring. As will be further explored in the data discussion, the psychological dimension of older people has also been affected by the significant change in care practices during the pandemic emergency.

Evidence also pointed out in media representations, which in the lockdown phase, supported and expanded narratives about the pandemic and the associated fears, shifting the focus from the risk factors, which at the time were still little known, to the social consequences, as well as on the target population (Poli, 2020). While the World Health Organisation (WHO) highlighted the danger to the older population, it also noted the cross-sectional nature of the risk across all age groups, including younger people (Kluge, 2020). The media and various opinion leaders described the entire over-65 population as particularly fragile, to be safeguarded and protected. This communication has partially challenged the successful ‘active ageing’ paradigm and emphasised its limits (Cappelato and Mercuri, 2021). A change in ageing representations, with different outcomes, has emerged. Following the health emergency, older people are perceived as the most affected by Covid-19 (Daoust, 2020); victims of dangerous underestimation risks, especially if they are not self-sufficient and residents of residential homes; depicted indiscriminately as fragile, vulnerable subjects to protect (Previtali et al., 2020). Thus, it was hypothesised that older people might experience a marked impact on their psychological and social wellbeing, in addition to the impact on their physical health.

In several national and international studies analysing the impact of Covid-19 on the older population, very different approaches and research aims have been pro-posed (e.g., on the economic dimension; on formal and informal care; on the quality of life; on the health condition; on local and national care policies; on welfare systems; on representations and perceptions of ageing; on the risks of social exclusion; on redefinitions of everyday life and practices; on emerging needs and requirements) (Lebrasseur et al., 2021; Li and Mutchler, 2020; De Pue et al., 2021; Krendl and Perry, 2021). Even if to a lesser extent, some studies have also examined the relational aspect that characterises the care bond within the family. It emerged that during the pandemic emergency, the family support system was disrupted, with changes in support methods, and that feelings of belonging were challenged. Although the importance of social bonding and support within the family did not change during the pandemic, it could no longer be experienced in the same way. The desire to be close to family members and support them conflicted with the risk of contracting the pandemic (Derrer-Merk et al., 2024). Settersten et al. (2020) suggested, in a thought piece, that the pandemic had made people aware of how their lives were interlinked. They proposed, using the life course approach, to understand the impact and dynamics of the Covid-19 pandemic on individuals, family relationships, and the population. These studies found support for the Covid-19 social connectivity paradox: the need for social connection while maintaining social distance.

Considering the role families had in Italy in guaranteeing care activities before and after the pandemic, this contribution intends to investigate the impact that this unprecedented experience had on informal care practices and also on intergenerational relationships, focusing specifically on the relational aspect such as, for example, the frequency of contacts between older parents (aged 65 and over) and their non-cohabiting children, only partly already explored in the literature, as pointed out in the research by Vergauwen et al. (2022). The pandemic not only affected health outcomes but also had far-reaching consequences for caregiving practices, family dynamics, and the way care was provided to vulnerable family members.

## 4 Materials and methods

The analysed data were selected from the open-source SHARE (Survey of Health, Ageing and Retirement in Europe) database. The panel survey studies, in a transnational context and with a multidisciplinary approach, the health, ageing and retirement issues in Europe. It was launched in 2004 in 11 European countries, targeting individuals over 50 years old, as a response to the growing challenges of an ageing population. To date, SHARE has conducted eight data collection campaigns (waves), covering all countries of the continental European Union, Switzerland, and Israel. SHARE data collection is based on Computer-Assisted Personal Interview (CAPI), as it also enables the performance of physical tests (Börsch-Supan et al., 2013). Over the years, the SHARE database gathered microdata on health, socio-economic status, social and family conditions of the target population involved to understand the effects of health, social, economic and environmental policies over the lifetime of European citizens in order to turn the challenges of an ageing population into opportunities and to provide policy makers with reliable information for evidence-based policies. To understand the evolution of care relationships in Italian households during the pandemic, three particular data sets were selected for the present contribution:

- Wave 7 (W7), data collection was conducted in 2017 (Börsch-Supan, 2022a; Bergmann et al., 2019);
- Corona Survey 1 (SCS1), the first cycle of SHARE Corona survey conducted be-tween June and September 2020 (Börsch-Supan, 2022b; Scherpenzeel et al., 2020);
- Share Corona Survey 2 (SCS2), the second cycle of the SHARE Corona survey conducted between June and August 2021, 1 year after the previous wave, to observe developments during the pandemic (Börsch-Supan, 2022c; Scherpenzeel et al., 2020).

Within the three data sets, only the cases related to the Italian context were selected (Country Code IT = 16). At an overall presentation, the selected samples were found to be similar and thus comparable, considering specifically the gender and age variable (Table 1).

**Table 1:** Sample of SHARE Wave 7, CS1, CS2 series data on Italy by gender and age group in frequency (*N*) and percentages (%).

Gender	W7		CS1		CS2	
	*N*	%	*N*	%	*N*	%
Male	2,049	44.9	1,743	44.4	1,474	43.9
Female	2,519	55.1	2,182	55.6	1,886	56.1
Total	4,568	100.0	3,925	100.0	3,360	100.0

Age	W7		CS1		CS2	
	*N*	%	*N*	%	*N*	%
Over 65 From 1919 to 1958	3,817	83.6	3,223	82.1	2,750	81.9
Under 65 From 1959 to 1979	751	16.4	702	17.9	610	18.1
Total	4,568	100.0	3,925	100.0	3,360	100.0

Sources: our elaboration from SHARE dataset (Börsch-Supan, 2022a–c).

In March 2020, due to the outbreak of Covid-19, the data collection campaign, which had started in October 2019, was suspended when approximately 70 per cent of all interviews planned for the panel sample in the various participating countries had been conducted. In order to continue the fieldwork, given the situation in the field, the survey was continued with telephone interviews (CATI—Computer Assisted Telephone Interview) using a specific questionnaire covering the same topics as the regular SHARE questionnaire, but considerably shortened and targeted at the Covid-19 living situation of people aged 50 and older (Scherpenzeel et al., 2020).

Specifically, the SHARE Corona Survey examines in greater detail health status, habits, mental health, Covid-19-related symptoms, healthcare, socio-economic changes, and social networks. Furthermore, considering the purpose of the contribution and the different questionnaires used between Wave 7 and the subsequent Corona Surveys 1 and 2, only the sections relating to health issues in social, relational, support and care aspects (Mental Health—MH; Social Support—SP; Health Care—HC) were included.^1^

The analysis is based on aggregated quantitative data expressed in terms of frequencies, drawn from the SHARE Wave 7, CS1, and CS2 datasets. Through a longitudinal comparison, the delta of the frequencies between the various measurements was calculated in order to assess changes over time and identify possible trends or dynamics.

The aim is to explore how the widespread disruptions caused by social, economic, and health crises have reshaped the caregiving experience within families, specifically through a relational lens. In times of instability, family care extends beyond mere physical assistance to encompass emotional support, role negotiation, and the restructuring of intergenerational dynamics. These disruptions have compelled family members to adjust to new caregiving roles, often blurring the lines between formal and informal care. The relational aspect of caregiving becomes particularly significant, as it highlights how family members negotiate, share, and redistribute caregiving responsibilities in response to external pressures. Through this lens, the caregiving experience is not seen as an isolated or individual task but as a shared, evolving process influenced by the broader societal context, ultimately transforming how family members relate to one another during times of crisis.

## 5 Results

### 5.1 The impact of restrictive policies on family interactions: between resistance, loneliness and new modalities

Before the pandemic crisis, studies showed that most older people in Europe had at least weekly contact with their non-cohabiting children (Steinbach et al., 2019). As expected, the new emergency and the related containment actions undertaken have inevitably had a significant impact on physical and in-person contacts. This challenged family equilibrium, wellbeing and quality of life in older people. Data on habits involving physical interaction has significantly decreased, if not stopped completely. Out of all the respondents to Corona Survey 1 (Table 2), 54 percent stated that they no longer visited their relatives, confirming the effectiveness of the restrictive policies (Börsch-Supan, 2022b).

**Table 2:** CAH011_ Since the beginning of the pandemic, how often have you performed the following activities compared to before (the outbreak)? ‘Visited other family members since the outbreak’. Answer modes: don’t know, often, sometimes, almost never or never. In frequency N and percentages %.

Answer modes	*N*	%
-2 No answer/-1 Do not know	6	0.3
1. Not any more	1,361	54.6
2. Less often	876	35.0
3. About the same	188	7.5
4. More often	32	1.3
5. Does not apply	32	1.3
Total	2,495	100.0

Source: our elaboration from SHARE dataset Corona Survey 1 (Börsch-Supan, 2022b).

A social change that, as emerged and confirmed in studies on the pandemic’s impact on the health condition of older people, also increased the risk of adverse phenomena such as, for example, depressive disorders, anxiety attacks and feelings of loneliness. This issue relates directly to the family network and the role it plays in reducing the older person’s perception of loneliness, where the expectations older people have of family members are fundamental. Expectations that assume specific connotations in different socio-cultural contexts, which, in the literature, are expressed through the concept of ‘loneliness threshold’.

**Figure 1:**
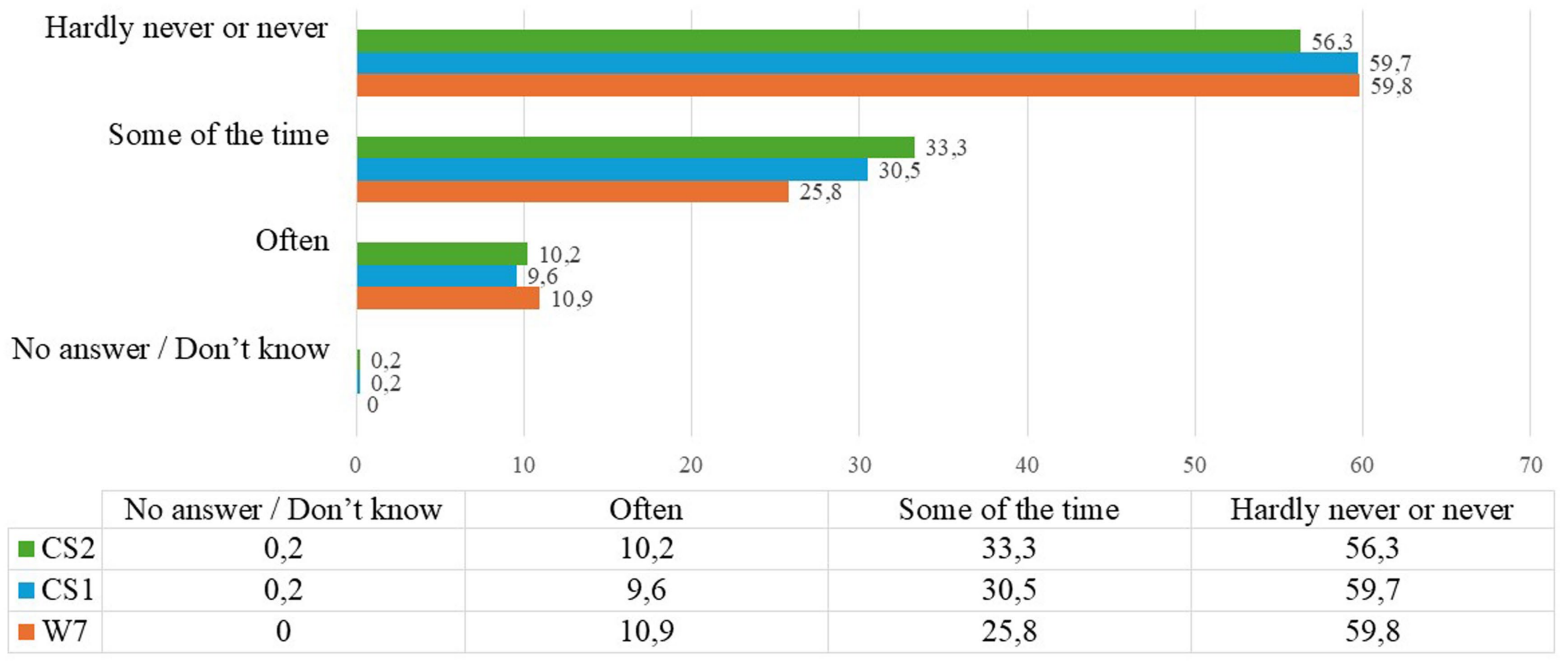
CAMH037 and MH037 how often do you feel lonely? Answer modes: do not know, often, sometimes, almost never or never. In percentage values %. Source: our elaboration from SHARE dataset Wave 7 (Börsch-Supan, 2022a); Corona Survey 1 (Börsch-Supan, 2022b); Corona Survey 2 (Börsch-Supan, 2022c).

During the pandemic, many older people, especially those who used to live alone in their own homes, suddenly found themselves without any support (often daily) and sometimes in complete loneliness, also due to the forced separation from their relatives, except for rare moments when food and medicines were delivered, when possible (Cerea, 2021). Also, because of the need for precautionary social isolation to avoid contagion, many families, due to fear, sacked their family carers or simply ‘sent them away’ if they were hired illegally. The analysed SHARE data concerning the ‘sense of loneliness’ in the pre- and post-pandemic context also show an increase in the intermediate positions, i.e., those who feel they are lonely ‘sometimes’, whose percentages range from 25.8 per cent in W7 to 33.3 per cent in CS2 (Figure 1).

However, if one looks at the variables related to interactions between the older people and family members and/or non-cohabiting acquaintances, these do not show a decrease, as one might expect, but rather a change and adaptation to the new scenario. As far as ‘face-to-face’ contacts are concerned, these occur mainly with sons/daughters; moreover, with respect to their frequency, the percentages increase significantly during the pandemic event, if we consider both ‘daily contacts’ (+10%) and ‘multi-weekly’ contacts (+10.6%). At the same time, though, ‘weekly’ interactions with relatives and non-relatives also show growth. This trend confirms how, even in the emergency, the entire family network is made up of one’s own children, but also of other relatives, friends, and neighbours who resiliently coped with the unchanged need for relationships and care.

An exciting aspect of this analysis also concerned the interaction modalities. As in many other areas, e.g., work and education, the obstacle of physical distance was overcome by new technologies that enabled the continuation of activities. In the new Share Covid Survey, the new modalities were therefore explored by distinguishing, unlike previous surveys, face-to-face contacts from long-distance contacts (by telephone, e-mail, or other electronic means). Here again, sons/daughters are the most frequent recipients of interactions. In CS1, ‘daily’ contacts account for 65.2 per cent, but in CS2, the percentage drops to 54.3 per cent (Table 3).

**Table 3:** CAS 004_Since the beginning of the pandemic, how often have you had contact by telephone, e-mail or any other electronic means with the following persons who do not live with you? CAS 104_During the last 3 months, how often have you had contact by telephone, e-mail or any other electronic means with the following persons who do not live with you? Answer modes: don’t know, daily, several times a week, about once a week, less often, never. In percentage values %.

Answer modes	Son/Daughter		Parents		Other relatives		Non relatives	
	CS1	CS2	CS1	CS2	CS1	CS2	CS1	CS2
-2. Refusal/-1. Do not know	0.3	0.5	3.1	1.8	0.6	0.5	0.5	0.4
1. Daily	65.2	54.3	35.6	26.9	10.2	7.4	7.4	6.4
2. Several times week	20.9	27.9	21.3	19.5	31.2	26.9	25.5	22.8
3. About once a week	3.9	6.6	4.7	9.5	24.5	27.3	23.9	24.7
4. Less often	3.0	4.5	6.1	6.7	24.7	29.3	28.6	33.6
5. Never	6.7	6.2	29.2	35.6	8.8	8.7	14.1	12.0
Total	100.0	100.0	100.0	100.0	100.0	100.0	100.0	100.0

Source: our elaboration from SHARE dataset Corona Survey 1 (Börsch-Supan, 2022b); Corona Survey 2 (Börsch-Supan, 2022c).

Another noteworthy trend concerns the frequency of weekly interactions with both relatives and non-relatives, which appears to have increased over time as digital technologies have become more widespread. As reported in previous studies (Arpino et al., 2020), digital tools may help isolated older adults cope more effectively with the pandemic by compensating for reduced physical interactions and fostering a sense of closeness and support from family members and friends. However, the digital divide continues to penalise those older people who lack access to adequate devices, internet connectivity, or digital skills.

From a longer-term perspective, this unequal access to digital resources may be theorised as a mechanism of cumulative disadvantage, with significant implications for intergenerational relations and individual wellbeing. Persistent digital exclusion risks increasing social isolation, reinforcing dependency on family caregivers, and limiting older adults’ autonomy in maintaining meaningful social ties. Conversely, when access and competencies are more evenly distributed, digitally mediated interactions can help sustain intergenerational solidarity over time. The long-term impact of the digital divide therefore, extends beyond communication practices, potentially shaping emotional wellbeing, caregiving dynamics, and the structure of family support networks.

### 5.2 Care practices and intergenerational support during the pandemic

The observed change in the quality and quantity of social interactions also relates to the issue of care and its practices. As confirmed in other research (Melchiorre et al., 2021, 2022), in these situations, the family still seems to be at the forefront of care activities and represents the ‘primary/dominant’ help, especially that given by children, but also by other relatives (grandchildren, siblings, cousins).

The intergenerational relationship is also built on the paradigm of mutual care and help.

**Figure 2:**
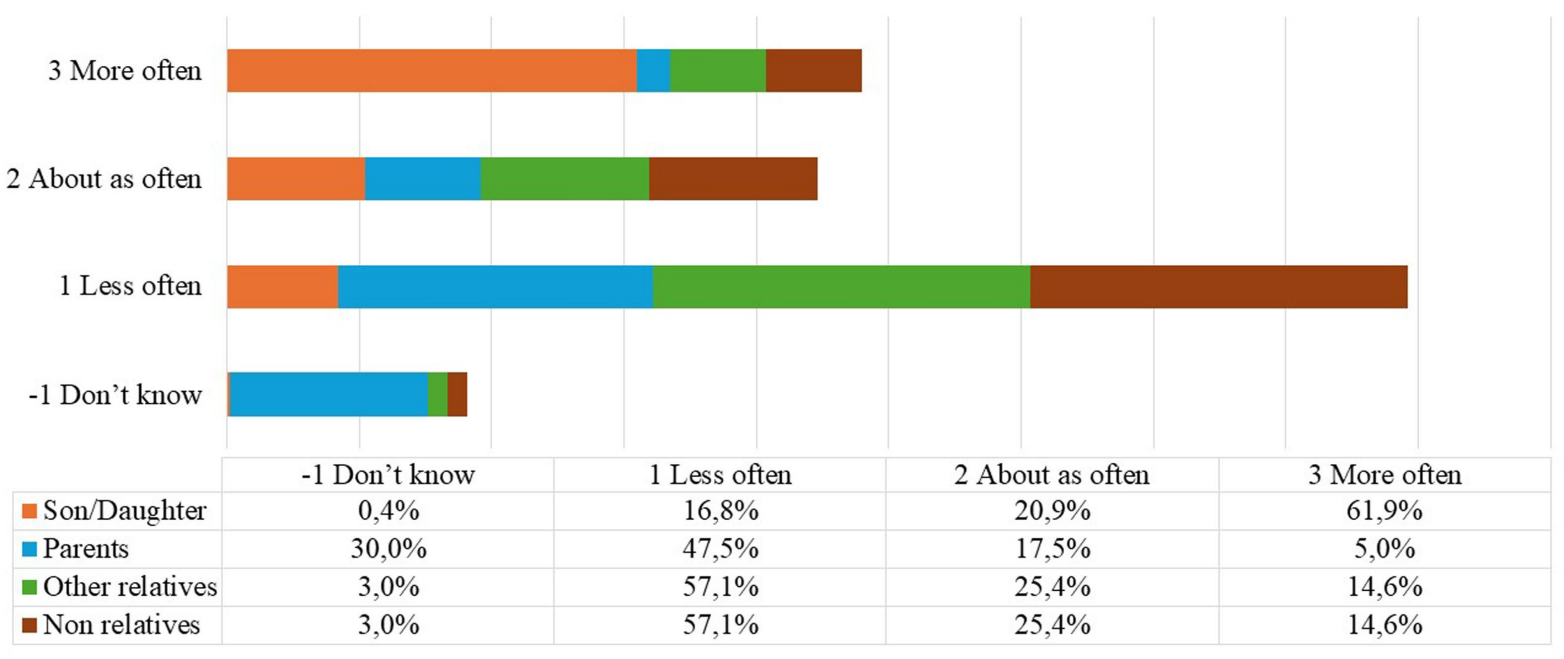
CS1_CAS021 Compared to before the beginning of the epidemic, how often did you receive help from the following people who do not live with you, to obtain essential goods or services? Answer mode: Do not know, less often, about as often, more often. Source: our elaboration from SHARE dataset Corona Survey 1 (Börsch-Supan, 2022b).

The extrapolated data concerning the help received and the help given to procure basic goods or services seem to confirm each other. In the first case (Figure 2), 61.9 percent of respondents stated that help from their own children increased (answer mode ‘more often’) compared to help received from other family members and non-family members.

**Figure 3:**
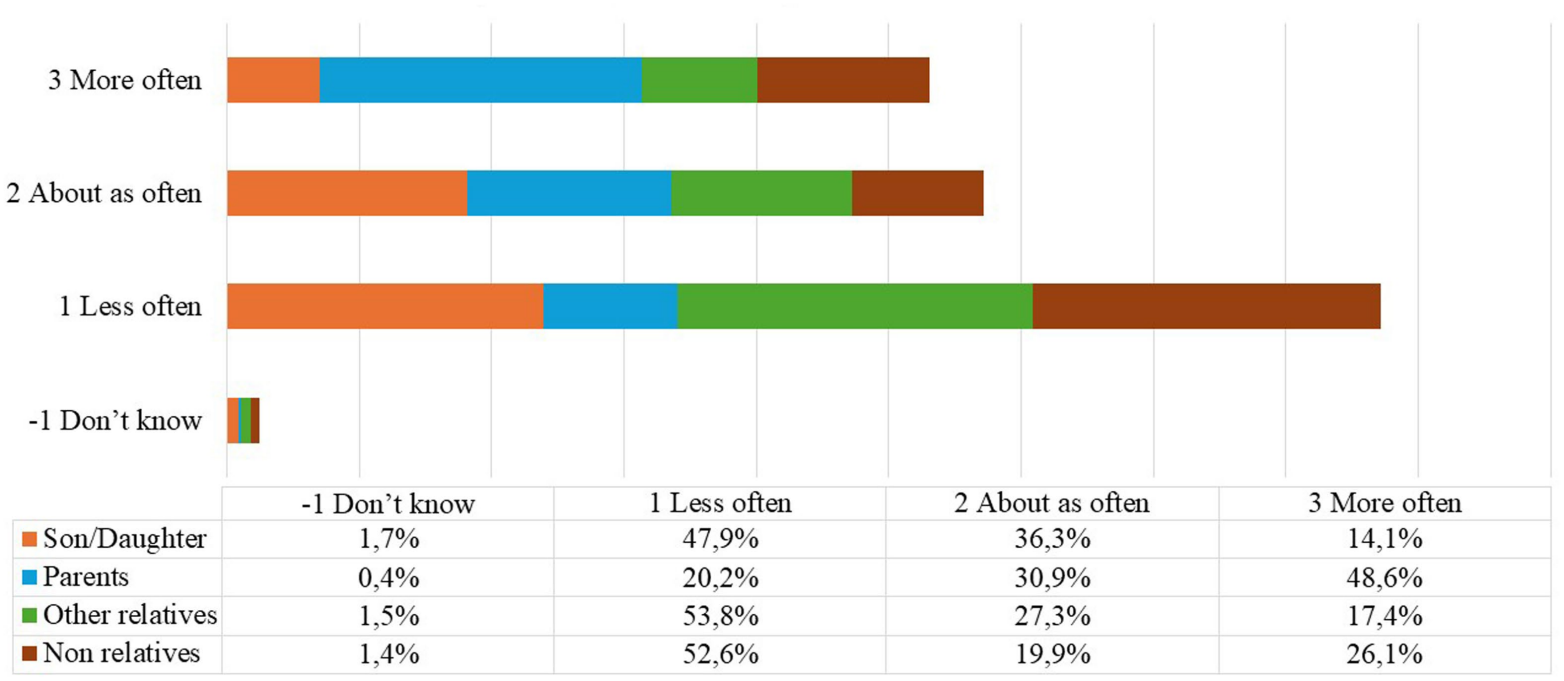
CS1_CAS011 compared to before the beginning of the epidemic, how often did you provide help to the following people who do not live with you, to provide them with essential goods or services? Answer mode: do not know, less often, about as often, source: our elaboration from SHARE dataset Corona Survey 1 (Börsch-Supan, 2022b).

Concerning the help provided, it is mainly women under65 who are engaged in personal care activities for non-cohabiting relatives. Furthermore (Figure 3), help given to one’s own parents is more frequent than to other possible recipients.

The data reveal a marked gender inequality in the intensification of informal support during the pandemic, both in material assistance and in personal care (Table 4). With regard to support aimed at accessing essential goods and services, a clear gendered division of labour emerges, with women disproportionately shouldering responsibility across almost all relational contexts. When considering only the response mode “more often,” women account for 60.6% of assistance provided to children and 66.1% of that provided to parents, underscoring their structural overrepresentation in the management of primary family needs. This pattern reflects the persistence of gendered expectations that position women as default providers of care and support within households. Although gender gaps are narrower in assistance to other relatives (52.2%) and to non-relatives (53.9%), women remain overrepresented, indicating that gender inequality extends beyond the nuclear family, albeit in more attenuated forms within extra-familial networks.

**Table 4:** Gender differences in the intensification of informal support and personal care during the COVID-19.

CAS_011_Compared to before the outbreak of Corona, how often did you help the following people from outside your home to obtain necessities: less often, about the same, or more often? Only response mode “more often.” In percentage values %.			
	Male	Female	Total
CAS_011_ 1 “son/daughter”	39.4	60.6	100.0
CAS_011_ 2 “parents”	33.9	66.1	100.0
CAS_011_ 3 “other relatives”	47.8	52.2	100.0
CAS_011_ 4 “non relatives”	46.1	53.9	100.0
CAS013_How often have you provided assistance with personal care to the following people who do not live with you, compared to before the start of the pandemic? Only response mode “more often.” In percentage values %.			
	Male	Female	Total
CAS_013_ 1 “son/daughter”	54.5	45.5	100.0
CAS_013_ 2 “parents”	34.5	65.5	100.0
CAS_013_ 3 “other relatives”	27.8	72.2	100.0
CAS_013_ 4 “non relatives”	50.0	50.0	100.0

Source: our elaboration from SHARE dataset Corona Survey 1 (Börsch-Supan, 2022b).

Gender inequality is even more pronounced in the sphere of personal care, a domain long-standing characterised by a feminization of responsibility and a disproportionate care burden borne by women. Women provide personal care more frequently to parents (65.5%) and, most notably, to other relatives (72.2%), highlighting a strong concentration of women in forms of care that are highly time-intensive, relational, and emotionally demanding. However, the observed gender differences do not reach conventional levels of statistical significance and should therefore be interpreted as descriptive patterns rather than evidence of systematic gender inequality.

This unequal distribution reflects entrenched gender norms and contributes to the reproduction of cumulative disadvantages for women, particularly in terms of time availability, emotional labour, and work–family balance. These trends are further supported by other studies, which show that the majority of informal caregivers experienced a significant increase in their caregiving responsibilities following the Covid-19 emergency, particularly in relation to the care of older adults. This highlights, on one hand, the gendered nature of caregiving, where women, more than men, tend to bear the brunt of these increased demands, while on the other, it emphasises the crucial role of family relationships in offering both emotional and practical/material support.

## 6 Discussion

### 6.1 From physical isolation to digital gaps: the post-pandemic social vulnerability of the elderly

During the Covid-19 pandemic, the restrictive measures imposed to contain the spread of the virus profoundly affected family relationship dynamics, particularly between older people and their non-cohabiting children. Data show a sharp interruption in regular physical contact; the majority of respondents to the first Corona Survey reported no longer visiting their relatives, confirming both the effectiveness (and the harshness) of the containment measures (Börsch-Supan, 2022b). This change fueled a widespread sense of loneliness and vulnerability among the elderly, particularly those living alone and deprived of the daily support they had become accustomed to De Pue et al. (2021) and Krendl and Perry (2021). The pandemic has had profound and lasting effects on the psychological and social wellbeing of older adults, with loneliness and vulnerability emerging as critical issues in the post-pandemic landscape. The enforced social isolation and disruption of routine family interactions during the health crisis severely undermined the social support structures that many older individuals rely upon. Studies indicate a marked increase in feelings of loneliness, with a shift in the distribution of responses towards more frequent experiences of isolation. This heightened loneliness is not merely a transient emotional state but is closely linked to broader vulnerabilities, including deteriorations in mental and physical health, and increased risk of depressive and anxiety disorders.

The concept of the “loneliness threshold” helps to understand how older adults perceive and tolerate social isolation differently based on cultural expectations and prior social conditions (Perlman and Peplau, 1981; de Jong Gierveld and Tesch-Römer, 2012). This threshold varies across cultural and individual contexts, shaping the degree to which older adults experience social isolation as distressing.

During the pandemic, many older individuals who previously maintained regular contact with family members found these expectations unmet, exacerbating feelings of abandonment and emotional distress. Particularly vulnerable were those living alone or without robust social networks, for whom the absence of daily support translated into practical challenges as well as psychological hardship.

Moreover, the pandemic highlighted the complex interplay between physical isolation and digital exclusion (Cerea, 2021). Faced with physical distancing and isolation, many families adopted technological solutions, such as phone calls, video conferencing, and messaging apps, to maintain a sense of closeness and continuity in their interactions. The pandemic not only disrupted traditional forms of in-person contact but also accelerated the digitalisation of family relationships, especially among older adults. While digital technologies offered alternative means of connection, many older adults faced barriers related to access, skills, and confidence, further deepening their sense of disconnection. The benefits were not equally distributed, revealing both the potential and limitations of technology in sustaining intergenerational bonds and underscored the need for more inclusive digital policies that consider the vulnerabilities of older populations.

The digital divide, marked by disparities in access to devices, internet connectivity, and digital literacy, meant that some older adults remained excluded from these new forms of connection. As highlighted in various studies (e.g., Arpino et al., 2020), digital communication cannot entirely substitute for the emotional and physical intimacy of in-person interactions, but it has played a vital compensatory role during a time of unprecedented disruption.

Consequently, the post-pandemic period demands renewed sociological attention to the structural and interpersonal factors that shape loneliness and vulnerability among the elderly. Addressing these challenges requires not only enhancing access to social and digital resources but also fostering community-based interventions that rebuild trust, reciprocity, and meaningful social ties, ultimately contributing to the resilience and wellbeing of older populations.

### 6.2 Family and beyond: the evolving role of informal support networks in times of crisis

The analyses also reveal signs of adaptation: while physical contact decreased, there was an increase in interactions, particularly with children, through alternative, often digital, means. This relational reorganisation, though forced, allowed for the maintenance and, in some cases, the strengthening of meaningful bonds, emphasising the central role of the family as a space of care, emotional support, and cohesion, even in contexts of extreme social isolation.

The Covid-19 pandemic posed unprecedented challenges to social support systems, particularly for older adults vulnerable to isolation. Despite restrictions on physical interactions, informal support networks comprising family members, friends, and neighbours demonstrated remarkable resilience. Data from the SHARE survey indicate that, contrary to expectations, interactions within these networks did not diminish but rather adapted in form and frequency. Face-to-face contact with children increased, with a notable rise in daily and multi-weekly meetings, underscoring the central role of offspring in the familial care structure.

Beyond children, other relatives and community ties also contributed to sustaining social cohesion and care provision, highlighting the multifaceted nature of informal support. This adaptability reflects the deeply rooted social and cultural expectations surrounding care and mutual assistance in many societies. Overall, the persistence and evolution of informal support during the pandemic emphasise its critical function in buffering the negative effects of social isolation and reinforce the importance of fostering strong community ties in times of crisis.

Informal social networks surrounding the family have played a crucial role in sustaining caregiving activities both during and after the Covid-19 pandemic, promoting resilience among older adults, as underscored in multiple studies (Kunz and Gummer, 2022). While families remain the primary locus of care, the support extended by friends, neighbours, and extended kin has proven essential in filling gaps left by formal care services, which were often limited or disrupted during the health crisis. These networks enhance families’ capacity to provide emotional, practical, and material assistance, creating a web of mutual aid that strengthens resilience in times of social disruption.

The pandemic highlighted that caregiving is not only an individual or nuclear family responsibility but also a collective effort embedded in broader community ties. Such networks also facilitate the sharing of information and resources, helping to mitigate isolation and vulnerability, especially among older adults. Moving forward, recognising and supporting these informal networks is vital to developing more inclusive and sustainable care systems that can adapt to future crises while promoting social cohesion and wellbeing across generations.

### 6.3 Gendered care in crisis: the unequal burden of informal caregiving during the Covid-19 pandemic

The Covid-19 pandemic not only intensified existing caregiving demands but also highlighted persistent gender inequalities within family care responsibilities. Numerous studies (e.g., Altieri and Santangelo, 2021; Yavorsky et al., 2021) highlight that women, particularly those under 65, disproportionately assumed increased burdens of informal care during the crisis, especially for non-cohabiting relatives such as elderly parents or disabled family members. This caregiving encompasses a broad spectrum of tasks, from assisting with daily activities to providing emotional support, which are often undervalued and uncompensated. The pandemic’s disruption of formal care services, such as day centres and home care, heightened reliance on informal networks, further intensifying women’s caregiving roles (Power, 2020).

This gendered division of labour not only reinforces traditional social norms surrounding females and domestic responsibility but also has tangible impacts on women’s wellbeing, labour market participation, and economic security (Craig and Churchill, 2021). For many women, increased care demands translated into reduced working hours or exit from paid employment, exacerbating existing gender gaps in income and career progression. Mental health consequences, including heightened stress, burnout, and anxiety, have been widely documented among female caregivers during and after the pandemic.

Furthermore, cultural expectations often place the primary caregiving role on women, which shapes family dynamics and limits opportunities for equitable sharing of care tasks between genders. Addressing these entrenched inequalities requires multifaceted policy approaches that promote gender equity in care, including expanding accessible formal care services, supporting caregivers, and enabling flexible workplace policies. Equally important is the cultural shift necessary to challenge normative assumptions about gender roles in caregiving and to foster shared responsibility within households. These findings resonate with Tronto’s understanding of care as a socially and politically structured practice, as well as with Finch’s notion of negotiated family responsibilities. Only through such systemic and cultural changes can the disproportionate care burden borne by women be alleviated, both in ongoing recovery and future crises.

## 7 Conclusion

This work, analysing the SHARE data in the Italian context, aimed to explore the effects of the pandemic on family care practices, paying particular attention to intergenerational relations. To improve welfare policies and systems, the adaptations, strategies and tools implemented within families to cope with the crisis were investigated, not only in terms of health. The data analysis highlights and confirms the complexity and the extent of the burden faced by family carers and care beneficiaries in relation to the unintended consequences of Covid-19 epidemiological control measures.

In Italy, given the welfare system’s structure, particularly one integrated with informal care, the family, through intergenerational ties grounded in care as a cultural value, still plays a central role in providing assistance to its most vulnerable members. This is a fundamental structural function that, despite the pandemic, has not been interrupted; instead, it has demonstrated strong resilience, adapting its dynamics and strategies to the new historical-social context. As noted in the findings, intergenerational interactions did not cease during the pandemic but instead found new ways to re-signify a forced physical absence as presence. The innovative use of technologies in areas previously little explored, particularly during the health emergency, is a testament to this, and it is not limited to the care context.

New technologies have profoundly transformed the quality of family relationships, going beyond mere increases in contact frequency. The ability to communicate in real time, share moments on digital platforms, and coordinate family activities at a distance has redefined how emotional bonds are built and maintained. However, these changes are not without ambivalence: on the one hand, they facilitate family cohesion, but on the other, they risk exacerbating digital inequalities and replacing direct contact with a “mediated” presence, an aspect whose implications for emotional and relational wellbeing remain largely unexplored.

An epochal change that, in imagining new practices, has preserved, if not increased, the frequency of interactions, particularly those between parents and children, and brought to light more or less known inequalities, such as the digital divide or the gender dimension. The disproportionate burden placed on women in informal caregiving roles during the pandemic further illustrates the persistence of gender inequality in the distribution of unpaid care work within families.

The analysis of the Italian family welfare model highlights its strongly familistic structure, which relies on the informal, often invisible contributions of women within the household. Although this model has provided a degree of social stability in the absence of widespread public welfare, it is now increasingly unsustainable in light of demographic changes, shifting gender roles, and labour-market transformations.

In light of these findings, the Italian welfare system faces an urgent need to move beyond its traditional familistic orientation by adopting policy measures that explicitly recognise, support, and redistribute caregiving responsibilities. At the national level, this implies strengthening public long-term care by more systematically integrating formal and informal care. Concrete policy interventions could include expanding home-based care services, formally recognising family caregivers through dedicated allowances and social security contributions, and developing gender-sensitive policies to reduce the disproportionate caregiving burden borne by women. Additionally, targeted investments in digital literacy and access to technological tools for older adults and caregivers are essential to prevent the amplification of existing social and territorial inequalities.

From a broader European perspective, the Italian case highlights challenges that are shared by many Southern and continental welfare regimes, where family solidarity continues to compensate for gaps in public provision. In this context, European social policy frameworks could play a crucial role by promoting common standards for long-term care, supporting cross-national learning on hybrid welfare models, and allocating resources—through instruments such as the European Social Fund Plus or the Recovery and Resilience Facility—to enhance community-based care infrastructures and digital inclusion initiatives. Coordinated European action could also contribute to the systematic recognition of informal care as a pillar of social protection, fostering more resilient and equitable welfare systems across member states.

The Covid-19 pandemic, while representing a profound social tragedy, has thus revealed both the strengths and the limits of family-centred care arrangements. If adequately addressed through innovative and evidence-based policies, the transformations observed during the pandemic may become an opportunity to rethink welfare systems in a more inclusive, sustainable, and gender-equitable direction. Only through an integrated approach—combining public policy intervention, technological innovation, and sociological research—will it be possible to effectively support families, protect vulnerable populations, and respond to the long-term challenges facing contemporary European welfare states.

## Data Availability

Publicly available datasets were analysed in this study. The data can be found at: Börsch-Supan (2022a,b,c).

## Footnotes

1 Specifically, in addition to the demographic variables, the following ones were analysed: sp008_ sp002_ (Wave7); cah010_ cah011_1 cah011_2 cah011_3 cah011_4 camh037_ camh837_ cas003_1 cas003_2 cas003_3 cas003_4 cas004_1 cas004_2 cas004_3 cas004_4 cas010_ cas011_1 cas011_2 cas011_3 cas011_4 cas012_ cas013_1 cas013_2 cas013_3 cas013_4 cas015_ cas016_ cas020_ cas021_1 cas021_2 cas021_3 cas021_4 cas025_cas026_ cas027_1 cas027_2 cas027_3 (CS1); cah110_ cah111_3 cah111_6 cah111_7 cah111_8 cah111_11 camh037_ camh148_ cas103_1 cas103_5 cas103_2 cas103_3 cas103_4 cas104_1 cas104_2 cas104_3 cas104_4 cas110_1 cas110_2 cas110_3 cas110_4 cas111_1 cas111_2 cas111_3 cas111_4 cas112_1 cas112_2 cas112_3 cas112_4 cas113_1 cas113_2 cas113_3 cas113_4 cas115_cas116_ cas120_1 cas120_2 cas120_3 cas120_4 cas121_1 cas121_2 cas121_3 cas121_4 cas125_ cas130_1 cas130_2 cas130_3 cas130_4 cas130_5 cas131_1 cas131_2 cas131_3 cas131_4 cas131_5 cas126_ cas127_1 cas127_2 cas127_3 cas127_4 cas127_5 (CS2).
